# A Three-Gene Classifier Associated With MicroRNA-Mediated Regulation Predicts Prostate Cancer Recurrence After Radical Prostatectomy

**DOI:** 10.3389/fgene.2019.01402

**Published:** 2020-02-04

**Authors:** Bo Cheng, Qidan He, Yong Cheng, Haifan Yang, Lijun Pei, Qingfu Deng, Hao Long, Likun Zhu, Rui Jiang

**Affiliations:** ^1^ Department of Urology, The Affiliated Hospital of Southwest Medical University, Luzhou, China; ^2^ Department of Gastrointestinal Surgery, The Affiliated Hospital of Southwest Medical University, Luzhou, China

**Keywords:** prostate cancer, radical prostatectomy, protein-coding gene classifier, clinical recurrence, biochemical recurrence, microRNA

## Abstract

**Background and Objective:**

After radical prostatectomy (RP), prostate cancer (PCa) patients may experience biochemical recurrence (BCR) and clinical recurrence, which remains a dominant issue in PCa treatment. The purpose of this study was to identify a protein-coding gene classifier associated with microRNA (miRNA)-mediated regulation to provide a comprehensive prognostic index to predict PCa recurrence after RP.

**Methods:**

Candidate classifiers were constructed using two machine-learning algorithms (a least absolute shrinkage and selector operation [LASSO]-based classifier and a decision tree-based classifier) based on a discovery cohort (n = 156) from The Cancer Genome Atlas (TCGA) database. After selecting the LASSO-based classifier based on the prediction accuracy, both an internal validation cohort (n = 333) and an external validation cohort (n = 100) were used to examined the classifier using survival analysis, time-dependent receiver operating characteristic (ROC) curve analysis, and univariate and multivariate Cox proportional hazards regression analyses. Functional enrichment analysis of co-expressed genes was carried out to explore the underlying moleculer mechanisms of the genes included in the classifier.

**Results:**

We constructed a three-gene classifier that included FAM72B, GNE, and TRIM46, and we identified four upstream prognostic miRNAs (hsa-miR-133a-3p, hsa-miR-222-3p, hsa-miR-1301-3p, and hsa-miR-30c-2-3p). The classifier exhibited a remarkable ability (area under the curve [AUC] = 0.927) to distinguish PCa patients with high and low Gleason scores in the discovery cohort. Furthermore, it was significantly associated with clinical recurrence (p < 0.0001, log rank statistic = 20.7, AUC = 0.733) and could serve as an independent prognostic factor of recurrence-free survival (hazard ratio: 1.708, 95% CI: 1.180–2.472, p < 0.001). Additionally, it was a predictor of BCR according to BCR-free survival analysis (p = 0.0338, log rank statistic = 4.51).

**Conclusions:**

The three-gene classifier associated with miRNA-mediated regulation may serve as a novel prognostic biomarker for PCa patients after RP.

## Introduction

Prostate cancer (PCa) is the second most common cancer in males worldwide, representing a serious public health issue. It was estimated that there was 164,690 new PCa cases and 29,430 deaths in the United States in 2018 (Siegel and Jemal, 2018).

Approximately 80% of all PCa cases are diagnosed as localized PCa, and radical prostatectomy (RP) remains the cornerstone of therapy for the localized disease. Although RP provides durable cancer control for some, one-third of patients will experience biochemical recurrence (BCR) after curative surgery. In addition, BCR has been associated with the development of castration-resistant PCa and distant metastases (Brockman et al., 2015). Therefore, there is a great need to identify prognostic biomarkers for PCa to guide treatment decision-making.

Although current clinical and pathological indicators such as Gleason score (tumor grade), cancer stage, and prostate-specific antigen (PSA) level have been the most reliable prognostic factors, they do not accurately predict the progression risk of individual patients (Cooperberg et al., 2015; Leapman et al., 2018). Recently, many PCa prognosis-related protein-coding gene classifiers have been developed, as reported in previous studies. Abou-Ouf et al. (2018) reported a 10-gene classifier with the ability to distinguish aggressive and indolent PCa within low- and intermediate- risk groups. Jhun et al. (2017) constructed a 49-gene signature based on the Gleason score to improve the prediction of recurrence as well as ML progression in PCa patients after RP. Long et al. (2011) developed a classifier for use following RP involving 10 protein-coding genes and two *microRNA* (*miRNA*) genes, which increased the prognostic accuracy based on formalin-fixed specimens. Shahabi et al. (2016) reported a novel gene -expression based classifier for patients with early-stage localized PCa after RP, which was constructed using agnostic approaches based on whole genome expression profiles to improve upon the accuracy of clinical indicators to stratify patients at risk of clinical recurrence. However, the upstream molecular mechanisms underlying these classifiers remain unclear (Long et al., 2011; Shahabi et al., 2016; Jhun et al., 2017; Abou-Ouf et al., 2018).

MiRNAs are small single-strand non-coding RNA molecules (18–25 nucleotides), which regulate gene expression mostly at the posttranscriptional level (Karen et al., 2014). They can bind to completely or partially complementary mRNA targets and induce gene silencing by mRNA degradation or translational repression (Zamore et al., 2000; Hudder and Novak, 2008). Many miRNAs themselves have been identified as biomarkers for predicting the prognosis of PCa patients after RP using regression analysis. Fredsoe et al. (2019) reported a five-miRNA model (miR-151a-5p, miR-204-5p, miR-222-3p, miR-23b-3p, and miR-331-3p) for predicting of BCR, which was verified as a significant predictor. Another five miRNAs (miR-30c-5p, miR-31-5p, miR-141-3p, miR-148a-3p, and miR-miR-221-3p) were validated as independent prognostic biomarkers for PCa (Zhao et al., 2019). Furthermore, Kristensen et al. (2016) developed a three-miRNA prognostic classifier (miR-185-5p, miR-221-3p, and miR-326) to predict BCR independently of routine clinicopathological variables. It has also been demonstrated that miR-21 was an independent prognostic factor for BCR in patients with a Gleason score of 6 (Melbo-Jorgensen et al., 2014). However, the mechanisms between the apparent prognostic roles of these miRNAs and PCa remain unclear.

Therefore, we need to pay more attention to miRNA mediated regulation of protein-coding genes when developing gene classifiers to achieve increased understanding of the underlying molecular mechanisms.

In the present study, we developed a prognostic protein-coding gene classifier associated with miRNA-mediated regulation by comparing PCa patients with a high Gleason score (≥8) versus a low Gleason score (≤6) PCa patients after RP from The Cancer Genome Atlas (TCGA) cohort (Geybels et al., 2016). The classifier was then verified in an internal validation cohort and an independent external validation cohort from the Gene Expression Omnibus (GEO) database. Functional enrichment analyses of co-expressed genes were conducted to reveal the downstream mechnisms underlying the predictive ability of the classifier.

## Materials and Methods

### Study Population

Gene expression and miRNA data and corresponding clinical information were obtained from the TCGA- prostate adenocarcinoma (PRAD) dataset using the UCSC Xena browser (https://xenabrowser.net/). TCGA data from 333 PCa patients who underwent RP were included in the present study. TCGA samples with a high Gleason score (≥8) (n = 129) or a low Gleason score (≤6) patients (n = 27) were used as the discovery cohort to construct a gene classifier (associated with miRNA-mediated regulation) for predicting prognosis. The entire set of TCGA samples (n = 333) was used as an internal validation cohort. Another cohort of 106 samples from 100 PCa patients after RP was obtained from the GEO database (accession number: GSE54460 (Qi et al., 2014)). Duplicates from six patients were removed, and the remaining 100 samples were used as the external validation cohort. The clinical characteristics of the PCa patients after RP in the discovery cohort, internal validation cohort and external validation cohort are summarized in **Table 1**.

**Table 1:** Clinical characteristics of the prostate cancer (PCa) patients after radical prostatectomy (RP).

Parameter		Discovery cohort (n = 156)		Internal validation cohort (n = 333)		External validation cohort (n = 100)	
Age at diagnosis (mean ± SD)		61.2 ± 6.6		60.7 ± 6.8		61.1 ± 6.6	
Clinical stage, n(%)	≤T2a	56	36%	147	44%	43	43%
	T2b	17	11%	35	11%	8	8%
	≥T2c	44	28%	76	23%	48	48%
	Null	37	24%	75	23%	1	1%
Pathological stage, n(%)	Local	35	22%	128	38%	–	–
	Regional	119	76%	200	60%	–	–
	Null	2	1%	5	2%	–	–
Gleason score, n(%)	≤6	27	17%	27	8%	11	11%
	7	0	0%	177	53%	75	75%
	≥8	129	83%	129	39%	14	14%
PSA at diagnosis (ng/ml), n(%)	0-3.9	126	81%	283	85%	9	9%
	4-9.9	5	3%	6	2%	58	58%
	10-19.9	3	2%	7	2%	19	19%
	≥20	3	2%	3	1%	11	11%
	Null	19	12%	34	10%	3	3%
Surgery margins, n(%)	Negative	–	–	–	–	56	56%
	Positive	–	–	–	–	39	39%
	Null	–	–	–	–	5	5%
RFS, n(%)	Yes	29	19%	37	11%	–	–
	No	108	69%	255	77%	–	–
	Null	19	12%	41	12%	–	–
BCR, n(%)	Yes	–	–	–	–	49	49%
	No	–	–	–	–	51	51%
	Null	–	–	–	–	0	0%

### Preprocessing of the Gene Expression and miRNA Data

For samples in the TCGA dataset, gene expression and miRNA profiles were converted to log_2_(x + 1) of the initial expression value. Only protein-coding gene profiles were extracted for further analysis by identifying genes with protein products in the HUGO Gene Nomenclature Committee (HGNC) database (https://www.genenames.org/). Given that failures of gene and miRNA expression detection might exist, we only selected genes and miRNAs that were abundantly expressed for further analysis. The criteria were as follows: (1) expression level >0; (2) appeared in >50% of all specimens. For samples in the external validation cohort (from the GEO database), the gene expression data were preprocessed into fragment per kilobase per million reads (FPKM) values.

### Statistical Analysis

We constructed a gene classifier associated with miRNA-mediated regulation using the following four steps. Step 1 involved obtaining candidate genes and miRNAs. Logistic regression was applied to the discovery cohort to identify candidate genes and miRNAs (with p < 0.05) associated with the Gleason score. Step 2 involved obtaining significantly negitively correlated miRNA-gene pairs. A Pearson correlation analysis was applied to each miRNA-gene pair based on the candidate genes and miRNAs identified in step 1. A miRNA-gene pair with a Pearson correlation coefficient < -0.4 and p < 0.05 was defined as having a statistically significant negative correlation and used for further study. Step 3 involved, obtaining the target genes associated with miRNA-mediated regulation. We retrieved the potential target genes of the significantly negitively correlated miRNAs from miRWalk 3 (http://mirwalk.umm.uni-heidelberg.de/). Target genes that overlapped, i.e., were also significantly negitively correlated genes, were employed to develop the classifier. Step 4 involved, least absolute shrinkage and selector operation (LASSO) regression and a decision tree to further narrow down the variables and create two candidate classifiers. The Glmnet package was used to perform logistic regression and LASSO regression while the rpart package was used to construct the decision tree, in R software version 3.4.0.

Receiver operating characteristic (ROC) curve analysis was applied to assess the classifier’s ability to distinguish samples with a high or low Gleason score. Time-dependent ROC curve analyses were applied to assess the classifier’s ability to predict the 3-year clinical recurrence-free survival (RFS) rate and BCR-free survival (BCRFS) rate, using the survival ROC package in R software. Area under the curve (AUC) values were calculated to estimate the prediction ability of the classifier or related clinical factors. The Youden index from the time-dependent ROC curve analysis was employed to define the cutoff to split samples into high- and low-risk groups. The RFS and BCRFS were compared between the two groups according to the Kaplan-Meier method with the log-rank test.

Based on a Pearson correlation analysis of the genes in the classifier, genes with an absolute Pearson correlation coefficient >0.4 were identified as co-expressed genes (Seon-Kyu et al., 2015). Gene Ontology (GO) and Kyoto Encyclopedia of Genes and Genomes (KEGG) pathway enrichment analyses of the co-expressed genes were conducted using ClusterProfiler with p < 0.01 as the cut-off criterion (Yu et al., 2012). A network that involved the above-mentioned overlapping miRNA-gene pairs was visualized using Cytoscape 3.5.1.

## Results

### Construction and Assessment of the Protein-Coding Gene Classifier in the Discovery Cohort

**Figure 1:**
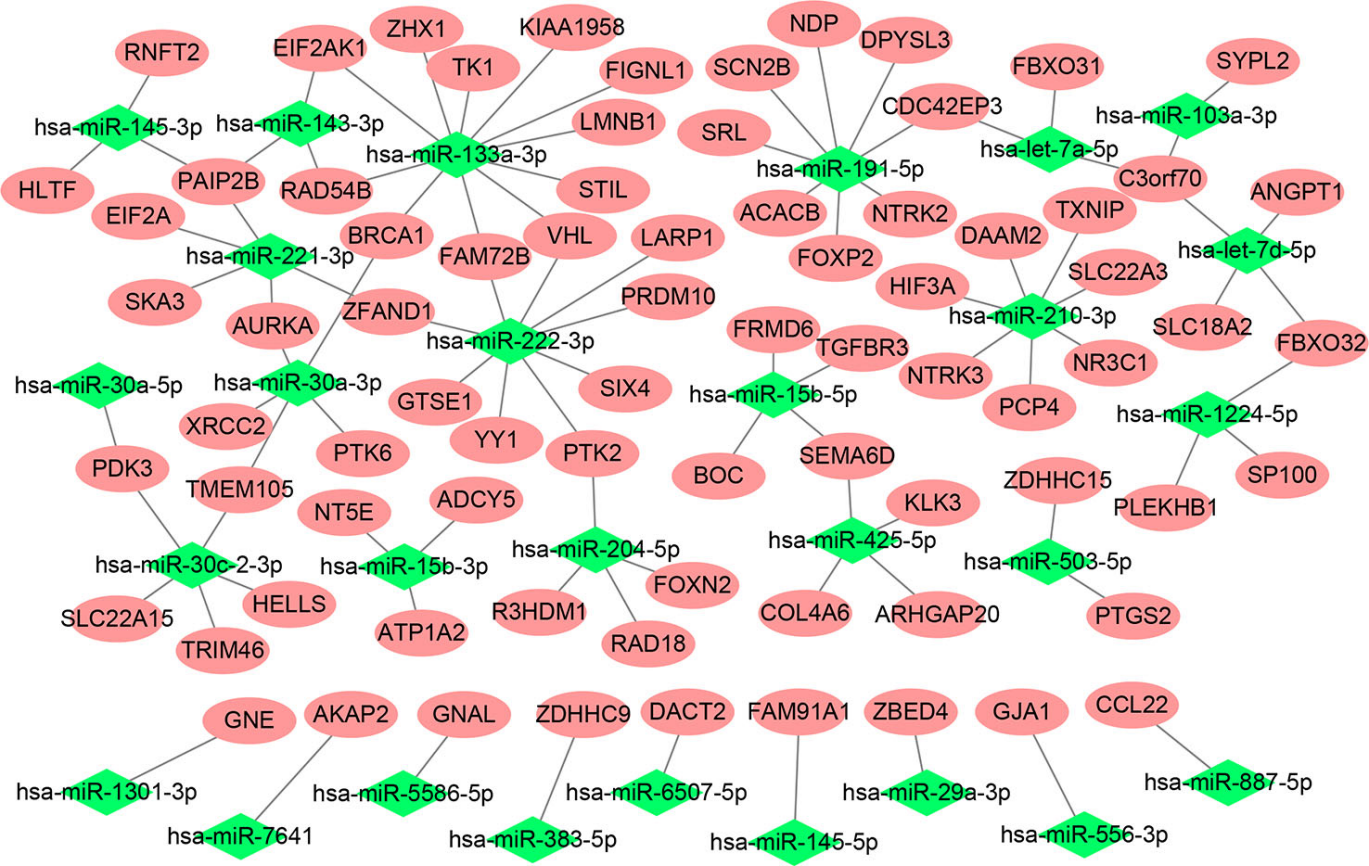
Network of the miRNA-gene pairs involving genes from both miRWalk 3 and the correlation analysis.

After preprocessing, each abundantly expressed gene and miRNA from samples in the discovery cohort was subjected to univariate logistic regression using binomial Gleason score as the dependent variable. Consequently, 3,732 genes and 98 miRNAs (**Additional File 1**) were found to be significant. Next, 1,235 significantly negitively correlated miRNA-gene pairs were identified using a Pearson correlation analysis (**Additional File 2**). Theredfter, we retrieved the potential target genes of the significantly negitively correlated miRNAs from miRWalk 3, obtaining 158,948 miRNA-gene pairs (**Additional File 3**). The miRNA-gene pairs involving genes that overlapped, i.e., were present in both sets (**Additional File 4**) were visualized in a network, with 79 genes and 28 miRNAs involved, as shown in **Figure 1**.

**Figure 2:**
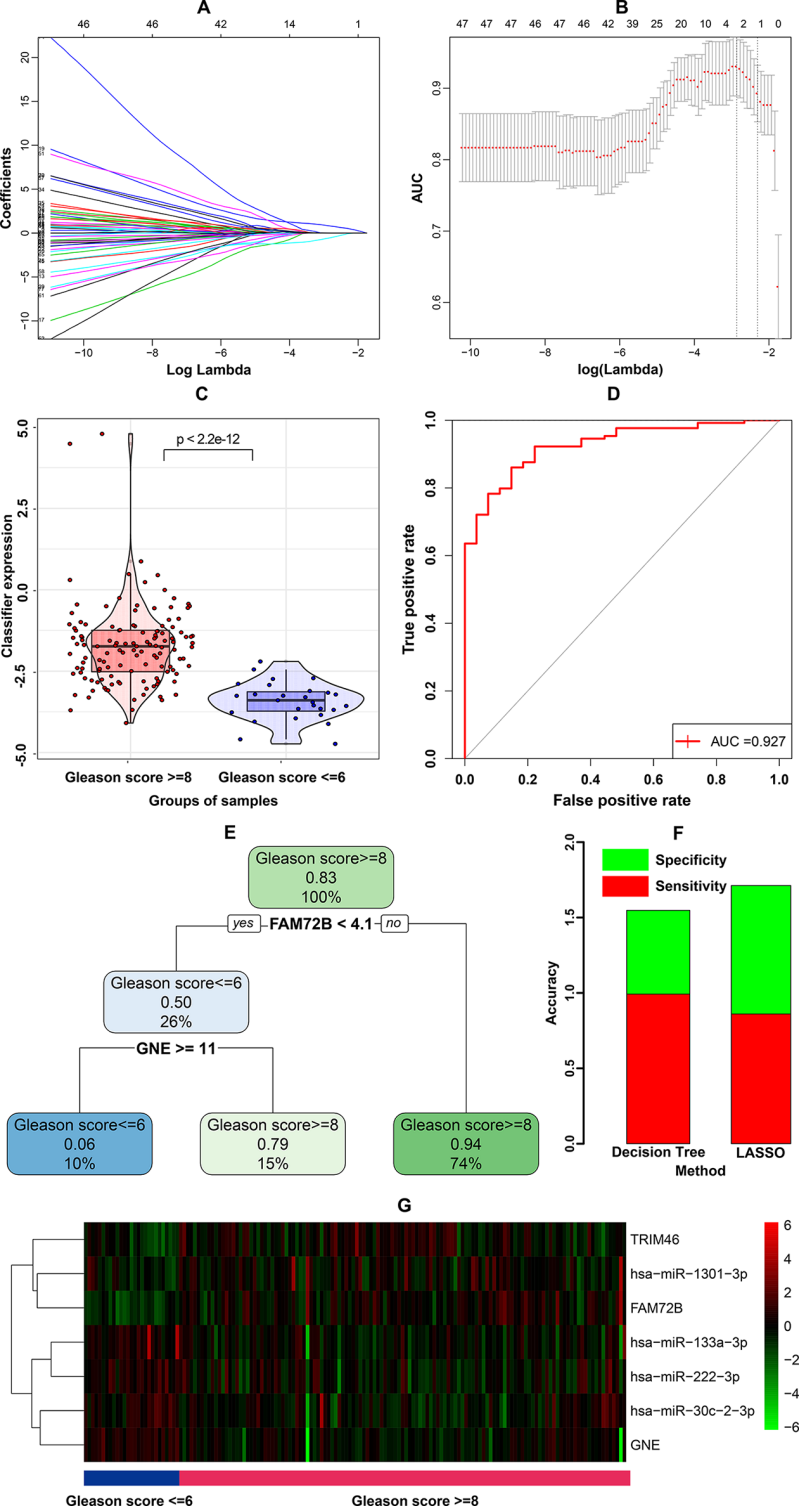
Construction and assessment of the three-gene calssifier associated with microRNA-mediated regulation. **(A)** Process of variable selection in least absolute shrinkage and selector operation (LASSO) regression. **(B)** Cross validation in LASSO regression. **(C)** Violin plot for the classifier. **(D)** Receiver operating characteristic (ROC) curve of the classifier’s ability to predict the Gleason score. **(E)** Decision tree based on the classification and regression tree (CART) algorithm. **(F)** Comparison of prediction accuracy between the LASSO-based classifier and decision tree-based classifier. **(G)** Heatmap of genes included in LASSO-based classifier and corresponding miRNAs.

These 79 genes were first subjected to LASSO regression, still using the binomial Gleason score as the dependent variable. LASSO regression is a parsimonious model that involves L1 regularization. λ is the coefficient of the penalty term in L1 regularization and as λ increases, the regression coefficients approach zero. Variables with non-zero regression coefficients are the variables most strongly associated with the dependent variable. Tenfold cross-validation was performed to determine the best λ value, with the AUC as the criterion. A series of models were constructed for variable selection (**Figure 2A**), among which the model with the highest AUC value was selected as the best model (**Figure 2B**). Three genes (FAM72B, GNE, and TRIM46) had non-zero coefficients in the best model. These three genes and their coefficients formed the following prognostic index (PI), representing a candidate classifier:

PI=(0.83444×FAM72B)+(−0.57533×GNE)+(0.01167×TRIM46)

The PI of samples were compared between groups with high and low Gleason scores using a violin plot, as shown in **Figure 2C**. To further evaluate the sensitivity and specificity of Gleason score prediction, we conducted a ROC curve analysis of PI. As shown in **Figure 2D**, PI exhibited a remarkable ability regarding Gleason score prediction (AUC = 0.927).

Furthermore, we used the 79 genes and the binomial Gleason score to construct a decision tree using the classification and regression tree (CART) algorithm. The decision tree with minimal cross-validation error was generated, as shown in **Figure 2E**, comprising two genes, FAM72B and GNE. As shown in the decision tree, FAM72B had almost complete dominance, while GNE came second. The coefficients in the PI indicated the same pattern. Therefore, the candidate classifiers obtained from the two machine-learning algorithms agreed well with each other, with the exception of TRIM46 in the LASSO-based classifier. To estimate the performance of the two classifiers, their sensitivity and specificity in classifying Gleason score were calculated, as shown in **Figure 2F**. The accuracy of the LASSO-based classifier was a little higher than that of the decision tree-based classifier. Thus, the PI derived from the LASSO-based classifier was identified as the final classifier.

After mapping the three genes in the classifier to the corresponding overlapping miRNA-gene pairs, we found that FAM74B was regulated by both hsa-miR-133a-3p and hsa-miR-222-3p, while GNE and TRIM46 were regulated by hsa-miR-1301-3p and hsa-miR-30c-2-3p, respectively. A heatmap of the expression of the three genes and four miRNAs was generated to gain more understanding of the correlations of individual genes and miRNAs with the Gleason score (**Figure 2G**). The heatmap shows that FAM72B, TRIM46 and hsa-miR-1301-3p were positively correlated with the Gleason score, whereas GNE, hsa-miR-133a-3p, hsa-miR-30c-2-3p, and hsa-miR-222-3p were negatively correlated with Gleason score.

**Figure 3:**
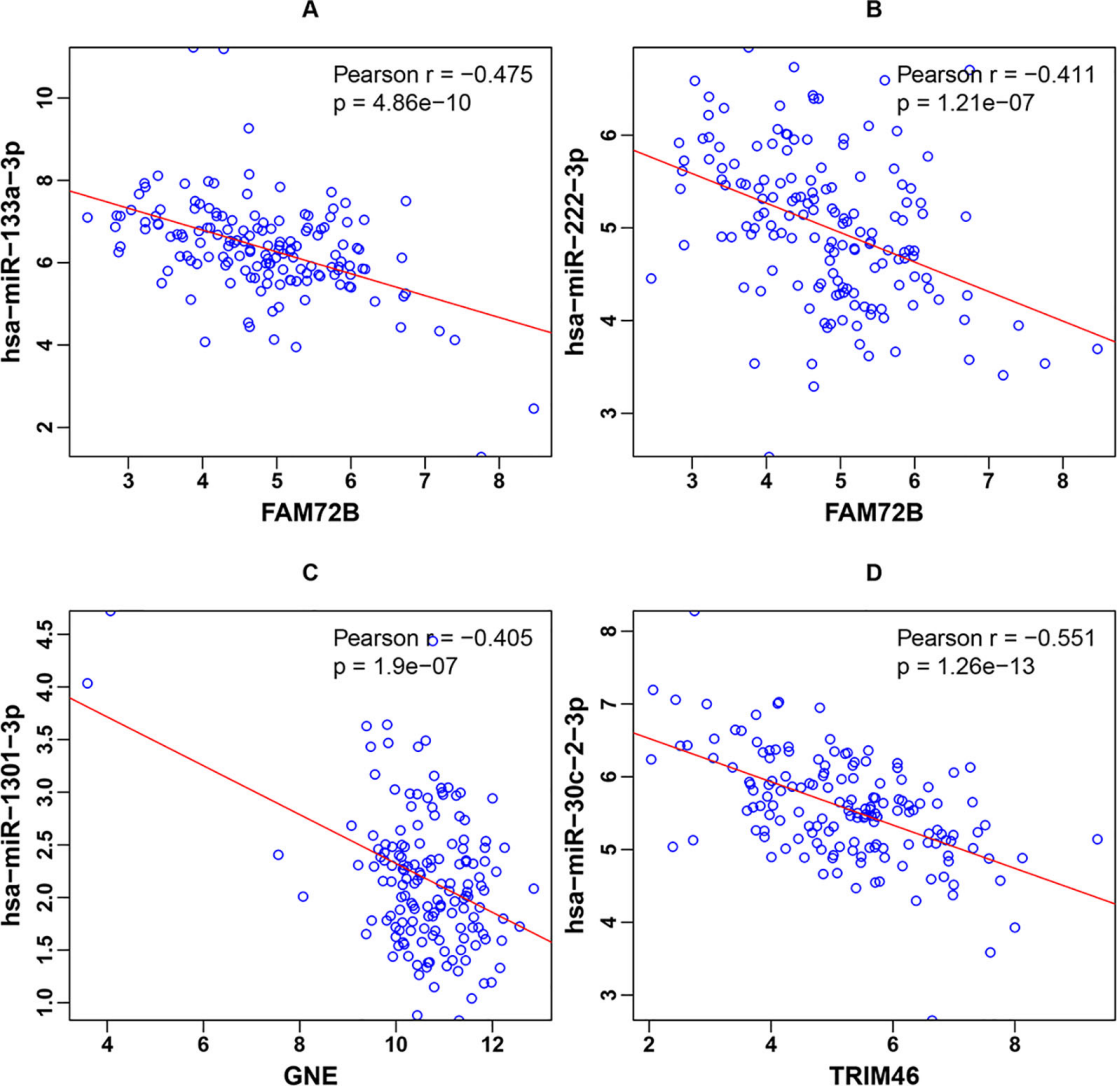
Scatter plots of the four miRNA-gene pairs’ expression and corresponding linear regression lines for **(A)** hsa-miR-133a-3p-FAM74B, **(B)** hsa-miR-222-3p-FAM74B, **(C)** hsa-miR-1301-3p-GNE, and **(D)** hsa-miR-30c-2-3p-TRIM46.

As described in the *Materials and Methods*, the three genes in the classifier were significantly negatively correlated with their corresponding miRNAs. Scatter plots of the four miRNA-gene pairs’ expression and corresponding linear regression lines were generated, as shown in **Figure 3**. The Pearson correlation coefficients were -0.475, -0.411, -0.405, and -0.551 for the miRNA-gene pairs hsa-miR-133a-3p-FAM74B, hsa-miR-222-3p-FAM74B, hsa-miR-1301-3p-GNE, and hsa-miR-30c-2-3p-TRIM46, respectively.

### Verification of the Protein-Coding Gene Classifier

**Figure 4:**
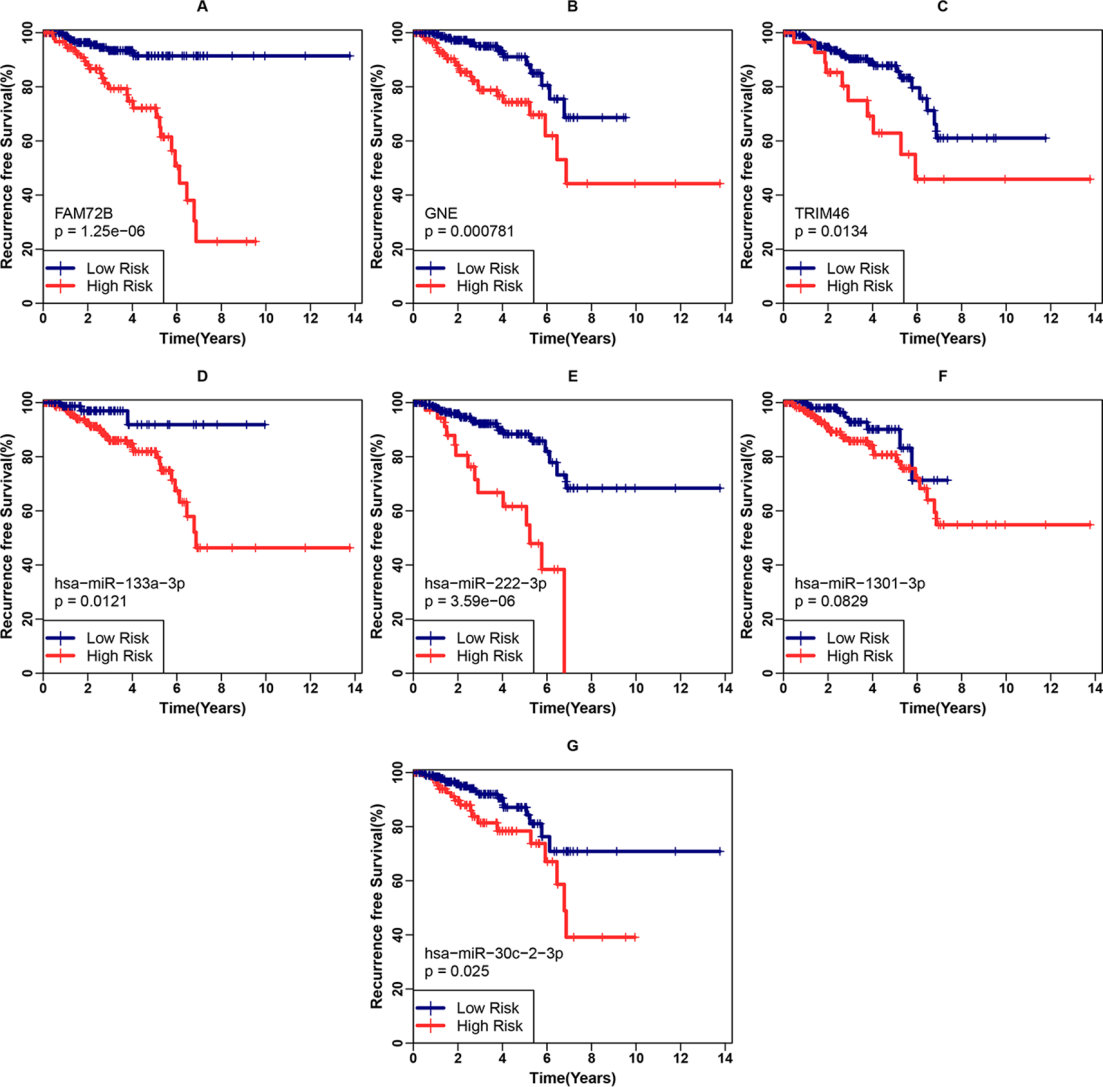
Examination of each gene included in the three-gene classifier and corresponding miRNA’s prognostic ability. **(A–C)** Recurrence-free survival (RFS) analyses for FAM74B, GNE, and TRIM46 in the internal validation cohort. **(D–G)** RFS analyses for hsa-miR-133a-3p, hsa-miR-222-3p, hsa-miR-1301-3p, and hsa-miR-30c-2-3p in the internal validation cohort.

First, we examined the associations between each gene included in the classifier and the clinical recurrence of PCa patients after RP in the internal validation cohort. RFS differences between the low- and high-risk groups were analyzed using the Kaplan-Meier method with the log-rank test, as shown in **Figures 4A–C**. We found that the three genes were all significantly associated with the RFS of PCa patients after RP (p < 0.001 for FAM74B, p < 0.001 for GNE and p = 0.0134 for TRIM46). Identical analyses for the four upstream miRNAs were also conducted, as shown in **Figures 4D–G**. Except for hsa-miR-1301-3p (p = 0.0829), all other miRNAs were significantly associated with RFS ((p-0.0121 for hsa-miR-133a-3p, p < 0.001 for hsa-miR-222-3p and p = 0.025 for hsa-miR-30c-2-3p).

**Figure 5:**
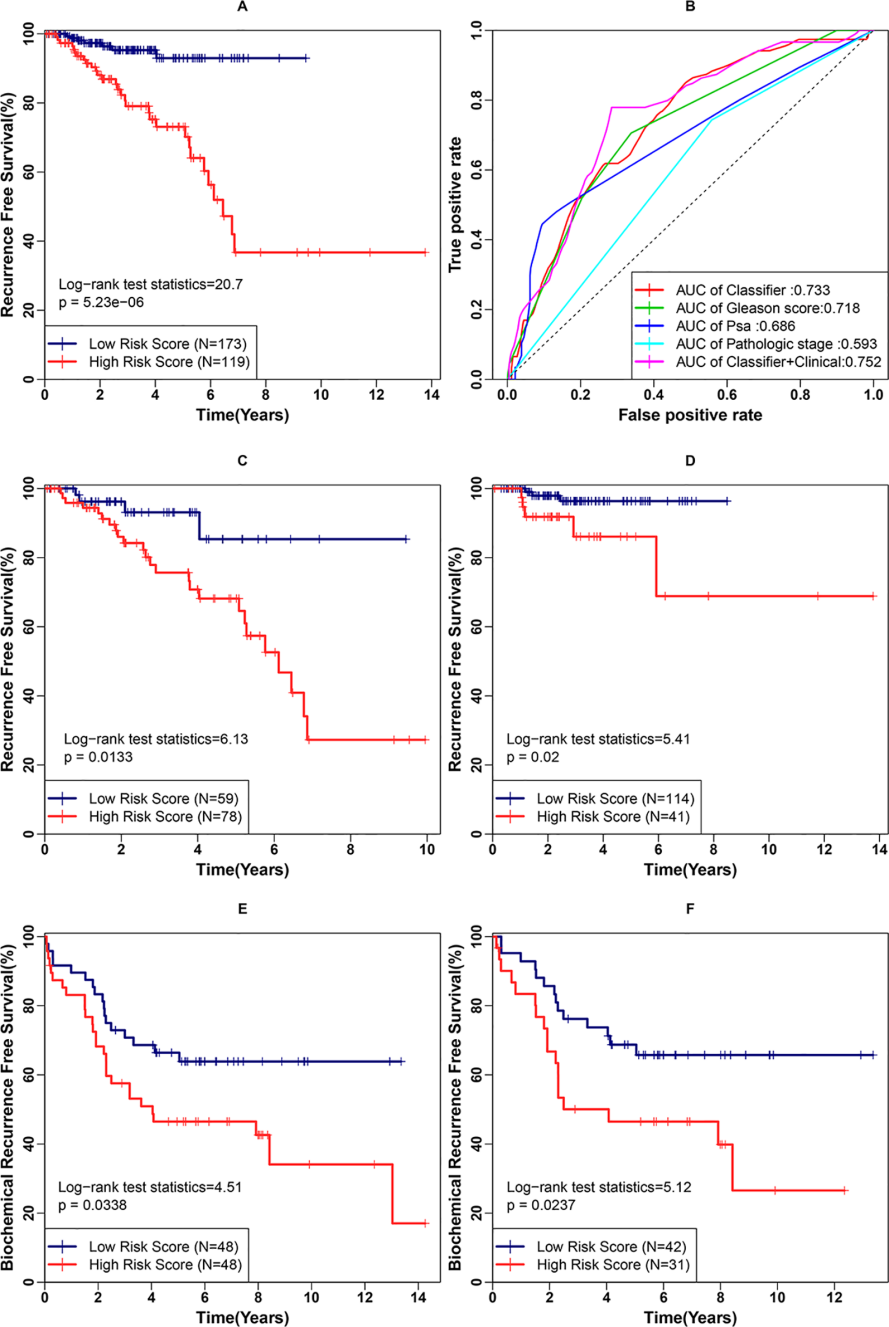
Examination of the three-gene classifier’s prognostic ability. **(A)** Recurrence-free survival (RFS) analysis for the classifier in the internal validation cohort. **(B)** Time-dependent receiver operating characteristic (ROC) curve analysis for the classifier and clinical factors in the internal validation cohort. **(C)** RFS analysis for the classifier in the discovery cohort. **(D)** RFS analysis for the classifier in the subgroup of the internal validation cohort with a Gleason score of 7. **(E)** BCR-free survival (BCRFS) analysis for the classifier in the external validation cohort. **(F)** BCRFS analysis for the classifier in the subgroup of the external validation cohort with a Gleason score of 7.

Second, we examined the classifier’s ability to predict clinical recurrence in patients after RP. With the use of the best cutoff point, the patients in this cohort were further divided into a high-risk group (n = 119) and a low-risk group (n = 173). The RFS difference was analyzed between the low- and high-risk groups (**Figure 5A**) in the internal validation cohort. The survival curves indicated a highly significant difference between the low- and high-risk groups (p < 0.001, log rank statistic = 20.7). To gain a deeper insight into the classifier, it was subjected to univariate Cox proportional hazards regression, as were four candidate clinical prognostic factors (age, Gleason score, PSA, and pathological stage) in the internal validation cohort. The results indicated that high classifier value, high Gleason score, high PSA value and Regional pathological stage were associated with significantly shorter RFS. The fmultivariate analysis confirmed that the classifier (hazard ratio [HR]: 1.708, 95%CI: 1.180–2.472, p < 0.001) and Gleason score (HR: 1.720, 95% CI: 1.089–2.716, p = 0.02) were independent risk factors for RFS. To further evaluate the sensitivity and specificity of the RFS survival prediction, we conducted time-dependent ROC curve analyses for the classifier and clinical factors. As shown in **Figure 5B**, the classifier exhibited a remarkable ability to predict RFS, as the AUC value was 0.733, which was higher than the AUC value for the Gleason score (0.718) (**Figure 5B**). Additionally, a combination of the classifier and three prognostic factors (Gleason score, PSA and pathological stage) achieved the best ability to predict RFS (AUC = 0.752). The results of the univariate and multivariate analyses of RFS in PCa patients after RP in the internal validation cohort are summarized in **Table 2**. Moreover, the classifier performed stably regarding risk stratification in both the discovery cohort (p = 0.0133, log rank statistic = 6.13) and the subgroup of the internal validation cohort with a Gleason score of = 7 (p = 0.02, log rank statistic = 5.41) (**Figures 5C, D**).

**Table 2:** Univariate and multivariate analyses of recurrence-free survival (RFS) in prostate cancer (PCa) patients after radical prostatectomy (RP) in the internal validation cohort.

Variable	Variable Treatment	Univariate				Multivariate			
		p	HR	95%CI(lower/upper)		p	HR	95%CI(lower/upper)	
Three-gene classifier	Continuous	**<0.001**	1.61397	1.33704	1.94826	**0.00458**	1.70751	1.17961	2.47166
Age	Continuous	0.30192	1.02595	0.97725	1.07707				
Gleason score	Continuous	**<0.001**	2.49847	1.74031	3.58692	**0.02005**	1.71988	1.08896	2.71633
PSA	Continuous	**0.02204**	1.05189	1.00731	1.09845	0.08653	1.04008	0.99438	1.08789
Pathological stage^a^	Binary (local VS regional)	**0.01417**	2.69347	1.22031	5.94504	0.79535	1.1207	0.47369	2.65146

a Local stage is pT2, N0/NX and M0. Regional stage is pT3-T4 and/or N1 and M0. The bolded texts have statistical significance.

Third, we examined the classifier’s prognostic ability to predict the BCR of patients after RP. The classifier’s output was calculated using the weight coefficients used in the above-mentioned prognostic index (PI). With the use of the best cut-off point, the patients in the external validation cohort were divided into a high-risk group (n = 48) and a low risk group (n = 48). The BCRFS difference was compared between the low- and high-risk groups (**Figure 5E**). The survival curves still indicated a significant difference (p = 0.0338, log rank statistic = 4.51). A similar result was obtained in the subgroup of the external validation cohort with Gleason score of 7 (p = 0.0227, log rank statistic = 5.12) (**Figure 5F**).

### Functional Enrichment Analysis of Co-Expressed Genes

**Figure 6:**
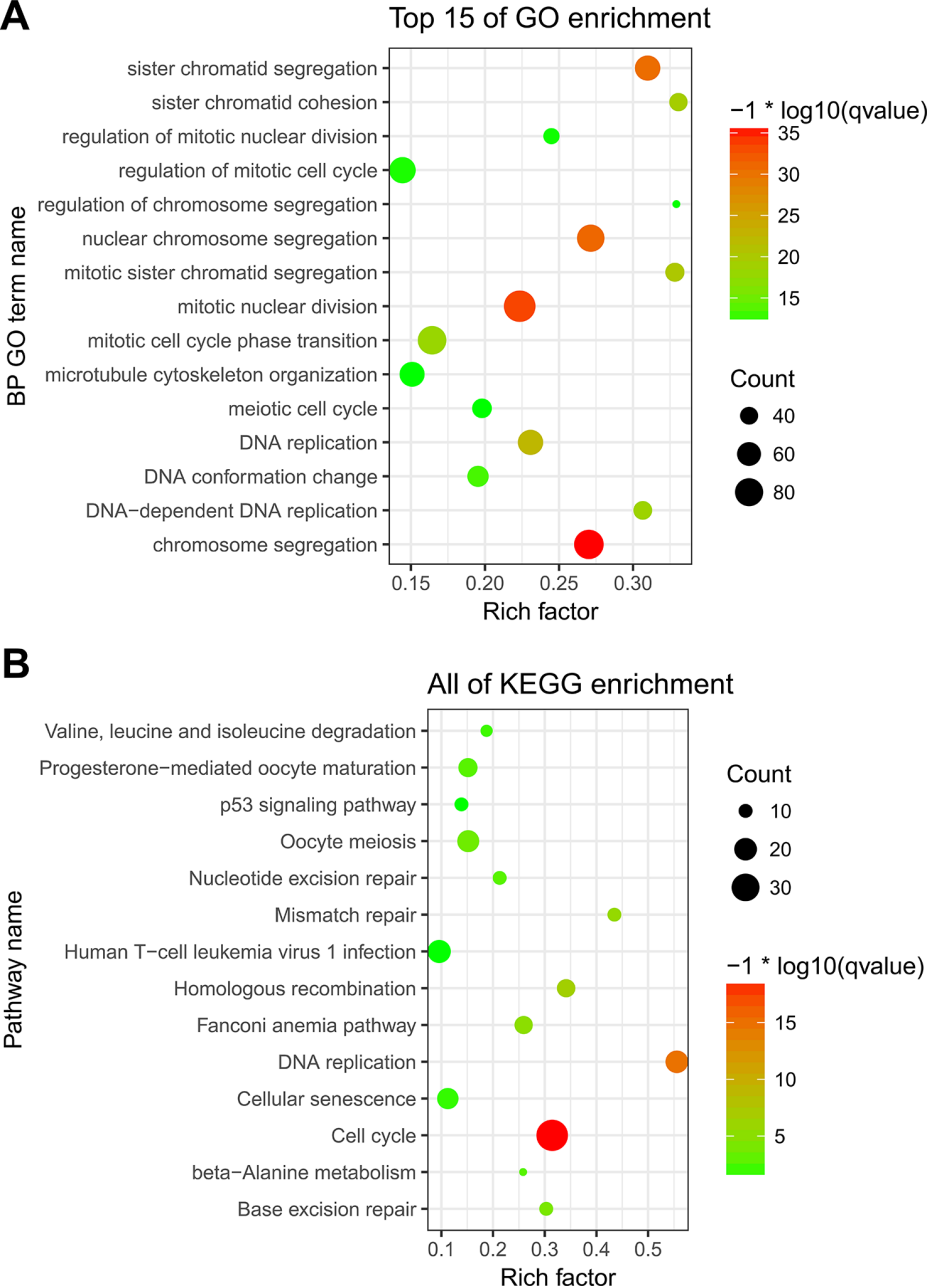
Functional enrichment analysis of the three genes’ co-expressed genes. **(A)** Significantly enriched Biological Process (BP) GO terms. **(B)** Significantly enriched Kyoto Encyclopedia of Genes and Genomes (KEGG) pathways.

To explore the underlying moleculer mechanisms of the three genes included in the classifier, we performed a functional enrichment analysis of the three genes’ co-expressed genes in the internal vlidation cohort. Genes with an absolute Pearson correlation coefficient >0.4 were identified as co-expressed genes. There were 673, 165, and 153 genes co-expressed with FAM72B, GNE, and TRIM46, respectively (**Additional file 5**). Finally, we obtained 894 co-expressed genes after removal of the duplicate genes. GO and KEGG enrichment analyses were conducted on these co-expressed genes, with Biological Process (BP) GO terms and pathways with p < 0.01 being defined as significantly enriched. The Top 15 of all 245 significantly enriched BP GO terms are visualized in **Figure 6A**, including chromosome segregation, mitotic nuclear division, nuclear chromosome segregation, sister chromatid segregation, DNA replication, and mitotic sister chromatid segregation. Significantly enriched KEGG pathways (n = 14) are shown in **Figure 6B**, including Cell cycle, DNA replication, Homologous recombination, Mismatch repair, Fanconi anemia pathway, Base excision repair, Oocyte meiosis, Nucleotide excision repair, Progesterone-mediated oocyte maturation and beta-Alanine metabolism, Valine, leucine and isoleucine degradation, Cellular senescence, Human T-cell leukemia virus 1 infection, and p53 signaling pathway.

## Discussion

In the present study, a three-gene classifier associated with miRNA mediated regulation was identified as a comprehensive prognostic biomarker that predicts both clinical recurrence and BCR for PCa patients after RP by comparing patients with a high Gleason score (≥8) to those with low Gleason (≤6) in a TCGA dataset and then validating the classifier.

The classifier, involving FAM72B, GNE, and TRIM46, showed a pronounced ability to predict the Gleason score of PCa patients after RP according to ROC analysis. Furthermore, both the three genes and the classifier showed remarkable prognostic ability to predict clinical recurrence in the internal validation cohort, and the classifier also had a ability to predict BCR in an indenpendent external cohort from the GEO database. It also performed stably regarding predicting prognosis in a subgroup of samples with Gleason score of 7 whose prognosis was difficult to predict (Geybels et al., 2016). Hence, the prognostic index (PI) based on the classifier derived in this study can act as a trustworthy index for clinical prognosis. In addition, four upstream miRNA (hsa-miR-133a-3p, hsa-miR-222-3p, hsa-miR-1301-3p, and hsa-miR-30c-2-3p) were identified as being both significantly negatively correlated with the three genes in the classifier and able to target the three genes, according to miRWalk 3. Three of the miRNAs (though not hsa-miR-1301-3p) were also prognostic biomarkers. Taken together, it is rational to hypothesize that the three genes in the classifer are regulated by these four miRNA, which may be a promising biological Research Topic.

Among the three genes, FAM72B and TRIM46 were correlated with a high Gleason score and a higher risk of clinical recurrence and BCR, whereas GNE was negatively correlated with the prognosis of PCa. It has previously been shown that FAM72B was differentially regulated following treatment with docetaxel chemotherapy and androgen deprivation therapy (ADT) in high-risk PCa patients, and it served as a prognostic biomarker (Rajan et al., 2014), which is in line with the present study. Notably, FAM72 has been reported as a novel neural progenitor cell (NPC) self-renewal supporting protein expressed under physiological conditions at low levels in other tissues and accumulating data indicate the potential pivotal tumorigenic effects of FAM72 (Kutzner et al., 2015). GNE is well known for its role in GNE myopathy, which is a rare muscle disease characterized by slowly progressive weakness and atrophy of skeletal muscles (Carrillo et al., 2018). A recent study demonstrated that GNE contributed to a strategy to provide novel insights into breast cancer subtypes and provide a foundation for new methods of diagnosis of breast cancer (Saeui et al., 2018). It has been found that TRIM46 was involved in the proliferation and migration of mouse and human breast cancer cells and TRIM46 could be inhibited by mmu-miR-1894-3p (Zhang et al., 2016). Furthermore, anti-TRIM46 antibodies have been found in patients with diverse neurological syndromes and are associated with small-cell lung carcinoma (van Coevorden-Hameete et al., 2017). TRIM46 also contributed to a classifier that identified subtypes of high-grade serous ovarian carcinoma (Kalpana et al., 2015).

Among the four upstream miRNAs, hsa-miR-133a-3p, hsa-miR-222-3p, and hsa-miR-30c-2-3p, but not hsa-miR-1301-3p, were significantly correlated with clinical recurrence. Recent studies showed that hsa-miR-133a-3p could serve as a diagnostic biomarker of rectal or colon cancer and also helped to diagnose and predict the prognosis of NSCLC (Wang et al., 2017; Weber et al., 2018). The Limited published literature related to hsa-miR-1301-3p, hsa-miR-222-3p, and hsa-miR-30c-2-3p can aid in understanding their functional mechanisms in PCa after RP.

Among the 14 significantly enriched KEGG pathways, cCell cycle (Tosoian et al., 2017; Léon et al., 2018), DNA replication (Tosoian et al., 2017), Base excision repair (Flores-Morales et al., 2018; Oing et al., 2018; Tonon et al., 2019), Nucleotide excision repair (Castro et al., 2015; Nordström et al., 2016), and the p53 signaling pathway (Bouali et al., 2008; Suk-Hyun et al., 2012; Gao et al., 2014) were extensively reported to participate in aggressiveness, growth, and metastasis of PCa after RP. Therefore, the classifier was evidently able to capture important biological pathways and events related to PCa. Our functional enrichment analysis results concur with previous research, and they also clarify the mechanisms underlying the prognostic relationship between the classifier and PCa outcomes after RP.

To our knowledge, the present classifier is the first protein-coding gene classifier associated with microRNA-mediated regulation to comprehensibly predict clinical recurrence and BCR for PCa patients after RP. In addition, we highlighted the prognistic roles of GNE and TRIM46, which have attracted some attention in previous studies.

The limitations of this study are as follows: (1) as our research is only based on analysis of secondary data, it is urgent to carry out biological experiments to verify our findings; (2) the gene expression data and clinical data employed in this study were obtained from open databases, so the quality of the data used cannot be fully evaluated; (3) other prognostic tools such as the Cancer of the Prostate Risk Assessment Post-Surgical (CAPRA-S) score and Decipher were not tested, so additional comparative studies are needed.

## Conclusions

In conclusion, we constructed a three-gene calssifier (involving FAM72B, GNE, and TRIM46) with comprehensive prognostic ability to predict both clinical recurrence and BCR for PCa patients after RP. The classifier’s prognostic mechanism may be associated with regulation mediated by four upstream miRNAs (hsa-miR-133a-3p, hsa-miR-222-3p, hsa-miR-1301-3p, and hsa-miR-30c-2-3p). These results provide guidance for PCa after RP and may help in patient management.

## Data Availability Statement

Publicly available datasets were analyzed in this study. This data can be found here: This data can be found here: https://xenabrowser.net/datapages/?cohort=TCGA%20Prostate%20Cancer%20(PRAD)&removeHub=https%3A%2F%2Fxena.treehouse.gi.ucsc.edu%3A443. GSE54460: https://www.ncbi.nlm.nih.gov/geo/query/acc.cgi?acc=GSE54460.

## Ethics Statement

All participants gave written informed consent. All authors have reviewed the manuscript and consented for publication.

## Author Contributions

BC, QH, YC, and HY contributed to the study design. LP and QD contributed to data collection. HL and LZ performed the statistical analysis and interpretation. RJ drafted the manuscript. All authors contributed to critical revision of the final manuscript and approved the final version of the manuscript.

## Conflict of Interest

The authors declare that the research was conducted in the absence of any commercial or financial relationships that could be construed as a potential conflict of interest.

## Abbreviations

AUC, area under curve; BCR, biochemical recurrence; BCRFS, biochemical recurrence-free survival; GEO, Gene Expression Omnibus; GO, Gene Ontology; KEGG, Kyoto Encyclopedia of Genes and Genomes; LASSO, least absolute shrinkage and selection operator; LR, logistic regression; PCa, prostate cancer; PI, prognostic index; PSA, prostate specific antigen; RFS, recurrence-free survival; ROC, receiver operating characteristic; RP, radical prostatectomy; TCGA, The Cancer Genome Atlas; 95% CI, 95% confidence interval.
